# One Sport or Many? Comparing the Effects of Athletics and Multiactivity Training on Motor Competence in 6–10-Year-Olds—A Case Study

**DOI:** 10.3390/jfmk10040479

**Published:** 2025-12-13

**Authors:** Nataniel Lopes, Miguel Jacinto, Diogo Monteiro, Rui Matos, Sérgio J. Ibáñez

**Affiliations:** 1 Facultad de Ciencias del Deporte, Universidad de Extremadura, 10071 Cáceres, Spain; nataniellopes@gmail.com; 2 Life Quality Research Centre (CIEQV), 2040-413 Leiria, Portugal; 3 Research Center in Sports, Health, and Human Development (CIDESD), 6201-001 Covilhã, Portugal; miguel.s.jacinto@ipleiria.pt (M.J.); diogo.monteiro@ipleiria.pt (D.M.); rui.matos@ipleiria.pt (R.M.); 4 School of Education and Social Sciences (ESECS), Polytechnic University of Leiria, 2411-901 Leiria, Portugal

**Keywords:** assessment, intervention, coordination, KTK3+

## Abstract

**Background**: Motor competence (MC) is defined as the ability to perform a wide range of motor skills with proficiency and control. The present quasi-experimental study design examines the impact of two structured intervention programs on MC in children who practiced athletics at the same club, aged 6 to 10 years, implemented over 12 weeks. **Methods**: The sample consisted of 64 children, assigned to two intervention groups: Intervention Group A (IG_A) composed of 15 male and 17 female children (9.57 ± 0.86 years) and Intervention Group B (IG_B), of 14 male and 18 female children (9.08 ± 1.33 years). IG_A received athletics-based training exclusively, three times per week, while IG_B undertook two weekly athletics sessions and one complementary activity session, such as handball, gymnastics, swimming, and motor games. MC was assessed using the modified Körperkoordinationstest für Kinder (KTK3+). The KTK3+ consists of three original KTK tasks, [Backward Balance (BB), Sideways Moving (MS), and Jumping Sideways (JS)] and an additional Eye–Hand Coordination (EHC) task. For statistical analysis, ANOVA repeated measures 2 × 2 was used. **Results**: In relation to JS, the performance on this test did not change with the intervention programs in either of the two groups. For BB and MS, both groups improved their performances in a similar way through the program implementation. Differently, for EHC, results showed that only IG_B improved its performance significantly (*p* < 0.001) with the program’s intervention, with a large Cohen’s d effect size (0.84). Finally, as a general analysis, the KTK3+ raw results (RS) and results translated to Global Motor Quotient (GQM), revealed significant differences between IG_A and IG_B post-intervention, with *p* < 0.001 for both variables’ comparison and with large Cohen’s d effect sizes for both (1.581 for RS and 1.595 for GQM), favoring IG_B. **Conclusions**: Both programs led to improvements in the various KTK3+ battery tasks. However, only the program that combined athletics training with multiactivity training led to significant improvements in the EHC test and in the overall KTK3+ results of the children involved.

## 1. Introduction

The period between 6 and 10 years of age represents a sensitive phase for the acquisition and refinement of fundamental movement skills (FMSs) [1], which constitute the building blocks of more complex actions and are predictive of lifelong motor proficiency [2,3,4,5]. FMS can be categorized into locomotor (e.g., running, jumping), stability (e.g., balancing, turning), and object control/manipulative skills (e.g., throwing, catching) [6].

Motor competence (MC), defined as the ability to execute a broad range of FMSs and other daily life motor skills with proficiency and control [7,8], plays a central role in children’s lives. Physical, cognitive, and socioemotional development, as well as psychosocial well-being and academic achievement, benefit from a good level of MC [4,9,10,11,12,13]. MC reflects multiple neuromotor mechanisms, including coordination, balance, and motor control [14,15], and serves as a foundation for physical activity (PA) engagement, sport participation, and the adoption of healthy lifestyle patterns across the lifespan [9,16], reinforcing its relevance as a multidimensional developmental construct. Conversely, children with lower MC tend to be more sedentary, report lower self-efficacy, and participate less in organized physical activities [17,18,19,20,21].

Athletics represents a sport inherently aligned with FMS, emphasizing running, jumping, and throwing. Despite this alignment, empirical evidence comparing athletics-based programs with more diversified training approaches remains limited. Lopes et al. [22] found that, within a group of 10-year-old children who practiced athletics, the ones with more practice years had significantly better MC. In addition, compared to children of the same age, Lopes et al. [23] identified significant differences in MC favoring 10-year-old children who practiced athletics when compared to children who played other sports. In both cases, children only performed one sport, leaving open the question of whether practicing more than one sport might be superior to practicing just one, namely athletics.

Indeed, studies conducted on diversified or multiactivity programs, including structured motor games [24,25,26], dance [11], exergames [12], and aquatic activities [27,28], revealed enhanced MC, even beyond the intervention period [29,30,31,32,33]. Additionally, other studies [34,35] argue that a more varied sports practice at young ages has a positive effect, among others, on gross motor function, i.e., better motor coordination and better MC. In a recent study with children aged 6 to 10 years old, Mancini et al. [36] concluded that participating in structured multisport programs appeared to enhance MC more than attending traditional school physical education. Barnett et al. [37], in a systematic review, highlighted the role of multisport programs in promoting MC. Some authors point out the importance of variability in practice to expand children’s motor repertoire [38]. This avoids the repetition of fixed actions, allowing participants to experiment with different ways of performing the same type of movement [39], contributing to the improvement of their motor skills. This diversified exposure has been linked to superior gains in MC levels when compared to single-modality training [31,32]. According to Luukkainen et al. [40], following a longitudinal study of the MC of children in Finland for three years (3–8 years old at time 1, 6–11 at time 2), a diversity of sport (multisports) participation allowed for better MC than participating in just one or not participating at all. Authors attributed the results to greater movement variety, potentially leading to enhanced MC. Moreover, authors stressed that this multisport participation did not necessarily imply engaging in multiple sports at the same time. It could happen over several seasons, and children still exhibited better skills resulting from their participation in more diverse sports. Thus, it may be the case that athletics training, often relying on repetitive, sport-specific movement patterns, may enhance mastery of targeted skills but, to some extent, limit exposure to varied motor challenges. In contrast, multiactivity programs integrating diverse movement experiences, such as gymnastics, swimming, handball, and motor games, may facilitate broader improvements in MC.

Building on these conceptual considerations and informed by a recent systematic review conducted by Lopes et al. [41], the present study compares the effects of two structured, coach-led 12-week training programs on children’s MC: (i) a program focused exclusively on athletics (IG_A), and (ii) a combined program (IG_B) integrating athletics with complementary activities designed to offer more diversified motor experiences.

Research gap and hypotheses. Despite the widespread use of athletics and the increasing adoption of multiactivity interventions, direct comparisons between these approaches to the promotion of MC remain scarce, making it unclear which program design more effectively promotes global MC gains in childhood. To address this gap, the present study compared the effects of a 12-week athletics-only program with a multiactivity program combining athletics with complementary activities. Thus, an attempt was made to determine whether modifying only one of the three weekly training sessions (i.e., replacing one athletics training session with a training session involving other motor activities) would lead to greater gains in terms of motor competence. Using the KTK3+ battery, we examined changes in MC in children aged 6–10 years.

The study tests the following hypotheses:

1. Both groups would show significant improvements over time in balance and locomotor-related tasks (BB, MS, JS), given that they would both have a core athletics training component where these types of tasks are central.
2. The multiactivity group (IG_B) would demonstrate greater improvements in the manipulative task—EHC, as manipulative tasks, namely object control, are less frequent in athletics training at these ages, and activities like handball or motor games were core to the multiactivity-specific practice of group B.
3. Global MC indicators (RS and GMQ) would improve more in the multiactivity group compared with the athletics-only group, because of the two previous hypotheses combined.

## 2. Materials and Methods

### 2.1. Study Design

This study followed a quasi-experimental design with parallel groups and was carried out according to the intervention protocol of Lopes et al. [42]. Participants were assigned to one of two intervention groups, following an ecological allocation procedure depending upon the training schedule chosen by their parents, according to their availability, with no prior indication of which of the two programs would function in which schedule, as stated in the referred Protocol Study [42]. The groups were (i) Intervention Group A (IG_A), which received athletics-based training exclusively, three times per week; and (ii) Intervention Group B (IG_B), which received two weekly athletics sessions and one complementary activity session (either gymnastics, handball, swimming, or motor games), maintaining the same weekly volume (3 sessions/week × 60 min). The participant flow for this implementation study followed the design outlined in the published protocol [42]. Briefly, children aged 6–10 were screened for eligibility and registered. Eligible participants underwent a baseline assessment (T0) and were then allocated to either Intervention Group A (IG_A) or Intervention Group B (IG_B) based on parental choice of training schedule. Both groups completed their respective 12-week structured training programs. A post-intervention assessment (T1) was conducted immediately after the program. Participants were excluded from the final analysis if they did not meet the inclusion criteria, declined to participate, failed to provide informed consent, or were excluded for other reasons (e.g., attendance below 80%).

The athletics sessions were conducted in the same setting for both groups by the same coach, the principal investigator of the present study. The handball, gymnastics, and swimming sessions were held during training hours in designated areas.

Furthermore, the study was conducted in an athletics training context. This choice was intentional, as the principal investigator is a qualified athletics coach and sought to explore whether a multiactivity approach could be meaningfully integrated within an athletics training framework without compromising the sport’s technical development. Given the central role of FMSs such as running, jumping, and throwing in athletics, it is crucial to assess whether complementing athletics training with activities from other areas can promote greater MC in middle childhood.

All the participants underwent MC assessments at two times: (i) at baseline (T0), prior to the beginning of the intervention; and (ii) at post-intervention (T1), immediately following the 12-week training period.

The post-intervention evaluations were carried out three days after the final training session, always in the afternoon, to ensure consistency in the testing conditions.

### 2.2. Participants

The sample consisted of 64 children (29 male and 35 female), 32 in each group (IG_A 15 male and 17 female (9.57 ± 0.86 years) and IG_B 14 male and 18 female (9.08 ± 1.33 years)), aged between 6 and 10, all of whom were enrolled in athletics when the study was conducted. It should be noted that all the children in the sample were members of the Federação Portuguesa de Atletismo and were all covered by sports insurance; therefore, they were protected in the event of an incident during data collection, which took place during training sessions.

To be eligible for inclusion in the study, participants needed to meet the following criteria: (i) be aged between 6 and 10 years; (ii) be officially registered in the Portuguese Athletics Federation; and (iii) have provided written informed consent signed by their legal guardians, along with the child’s verbal assent. We excluded from our sample children: (i) diagnosed with any physical or intellectual disability; (ii) with medical contraindications to physical exercise; (iii) who failed to obtain parental consent; (iv) who failed to complete the entire assessment protocol; or (v) falling outside the stipulated age range. Children meeting all eligibility criteria were ecologically assigned to one of the two intervention groups, as described in the study design: (i) Intervention Group A (IG_A) and (ii) Intervention Group B (IG_B).

Sample size and power calculations were performed using G*Power (v.3.1.9.7) [43]. Considering the analysis to be performed on the outcomes, a between–within ANOVA-RM (2 [groups] × 2 [time points]), anticipating a “large” effect size (f = 0.4), with an α = 0.05, a statistical power of (1 − β) = 0.95, the correlated dependent variables with a r = 0.50, and a violation of sphericity (ε) = 0.80, required a total sample size of 18 individuals. The suggested effect size and remaining parameters were defined according to similar studies [44,45,46].

### 2.3. Informed Consent

At an initial meeting, a comprehensive explanation of the study (including materials and methods) was presented by the research leader and the host institution to ensure that participants/family members/guardians were fully informed. In this meeting, all information about the distribution of children and the composition of the groups was also provided. A time was set for parents to decide on their participation by signing the informed consent form. By that time, all parents had signed the informed consent.

### 2.4. Motor Competence Assessment

To assess MC, several standardized tools have been developed, such as the Movement Assessment Battery for Children (MABC) [47], the Körperkoordinationstest für Kinder (KTK) [48], or the Motor Competence Assessment (MCA) [49], each capturing different aspects of gross motor competence. More recently, adaptations have been introduced to provide more comprehensive evaluations. Among these, the KTK3+, an expanded version of the KTK3, itself already an adaptation of the original KTK [50], incorporating an Eye–Hand Coordination (EHC) task [51,52], offers an integrated assessment of balance and stability skills and object control [51,53]. Thus, the present study employed the KTK3+ as an MC assessment tool, as it represents a more holistic and ecologically valid tool, aligning with theoretical frameworks that define MC as comprising locomotor, stability, and manipulative components [7,8,51].

The use of KTK3+ was therefore methodologically strategic, as it allowed us to comprehensively assess the multidimensional impact of both monosport and multiactivity training programs on children’s MC.

### 2.5. KTK3+ Test Application and Procedures

Regarding KTK3+ administration, the assessment team was composed of members who were external to the Athletics Club where the intervention took place (master’s students in the field of sports), and it was the principal investigator of the study who trained these members to collect data. This group of students already had some experience with three of the four KTK3+ tests, as they had integrated other research experiences using KTK and MCA (the latter sharing two of the four tests that compose KTK3+, namely MS and JS). Thus, the only new task for them was the EHC. Regarding this test, it is worth highlighting the clarification of doubts provided by the principal investigator of KTK3+, Prof. Dr. Sebastiaan Platvoet, regarding the particularities of applying this Eye–Hand Coordination test. At the time of the two assessments, the assessment team was unaware of the groups to which the different subjects belonged.

The test comprises four tasks: Task 1, Balancing Backwards (BB); Task 2, Jumping Sideways (JS); Task 3, Moving Sideways (MS); and Task 4, Eye–Hand Coordination (EHC) [54,55,56].

In the BB task, with three trials per balance beam, which decreases in width as the test progresses (6.0 cm to 4.5 cm to 3.0 cm), subjects must walk backwards over the beams. The maximum number of steps that can be performed on each beam is eight. In each trial, the count is interrupted whenever the subject loses balance and touches the ground with one foot. On each beam, the subject must perform three attempts. Thus, the maximum number of steps in this task, which will correspond to the test raw result, is 72.

In the JS task, participants had to jump with two feet over a wooden slat for 15 s. The final score resulted from the sum of the number of jumps in both trials.

In the MS task, participants had to move sideways in a straight line, handling two wooden platforms for 20 s. The total score results from summing the number of times participants put down a wooden platform, as well as the number of times participants stepped on the displaced wooden platform during both trials.

The EHC task is a valid and reliable product-oriented test [51] that determines the level of controlling a tennis ball while conducting repetitive movements (i.e., left hand throw, right hand catch, followed by right hand throw, and left hand catch, etc.) as frequently as possible in a time-constrained task of 30 s [52]. The participants were free to use overhand and/or underhand techniques or a combination of both for throwing and catching. For this purpose, participants had to stand 1 m from a wall and throw the tennis ball at eye-level in a square (1 m^2^) taped on the wall with the bottom side of the square 1 m above the ground. Participants performed this test twice, with the number of successful balls caught across both trials resulting in the test score.

The Raw Score (RS) of each task was compared with the normative values for that task and then converted into a motor quotient (MQ). The four motor quotients thus determined (one for each of the four KTK3+ tasks) were then summed to find the overall motor quotient (OMQ) obtained by each subject in the battery in question [51].

### 2.6. Intervention Protocols

Intervention Group A (IG_A) received a structured athletics-only training program, delivered three times per week for 12 consecutive weeks. Each session lasted 60 min and included drills and exercises focused on running, jumping, and throwing techniques, emphasizing the development of FMS within an athletics context (Appendix A). Intervention Group B (IG_B) undertook a multicomponent training program, consisting of two weekly athletics sessions identical to IG_A, and one additional 60 min session per week involving complementary activities (gymnastics, handball, swimming, or motor games). These additional sessions were designed to stimulate a broader range of motor abilities through exposure to diverse task demands, promoting adaptability and motor transfer (Appendix B).

The structure of each athletics session, for both groups, followed a consistent format: (i) warm-up (5 min): dynamic stretching or light jogging; (ii) main phase (45/50 min): focused athletics training targeting specific objectives—technique, speed, strength, and endurance; (iii) cool-down (5 min): static stretching or light jogging.

Despite all athletes (from 6 to 10 years old) belonging to the same competitive age group (“Benjamins”), which was an eminently formative and educational context, care was always taken to adjust, when necessary, the proposed exercises to the ages and abilities of the athletes (e.g., barrier height). In addition, each athlete performed the requested tasks precisely in accordance with their momentary capacity.

All sessions were supervised by coaches certified in the intervention protocols. A total of 6 coaches were involved in the two intervention programs: two athletics coaches (one responsible for IG_A and IG_B athletics training sessions—the principal investigator of the present study—and the other responsible for the motor games sessions on IG_B), two swimming coaches, one handball coach, and one gymnastics coach. The principal investigator was present to attend all sessions, having asked the coaches to ensure that the athletes experienced various basic technical aspects of these sports and activities. Prior to this intervention by these coaches, the researcher held meetings with them where he informed them of the program and asked them to do the above. In terms of attendance, 28 children (87.5%) in group A participated in 91.7% of training sessions and 4 (12.5%) participated in 88.9%, while in group B, 29 children (90.6%) participated in 91.7% of training sessions and 3 (9.4%) participated in 88.9%.

### 2.7. Statistical Analysis

Means and standard deviations were calculated for all studied variables. Normality and homoscedasticity were verified with the Shapiro–Wilk (*n* < 50) and Levene’s tests, respectively. Next, a within–between ANOVA repeated measures 2 × 2, (2 [groups] × 2 [time points]) was conducted to examine differences in dependent variables. For all tests, the significance level to reject the null hypothesis was set at 5%. Sphericity assumptions were examined using Mauchly’s test. When this assumption was not met, the Greenhouse–Geisser adjusted values and degrees of freedom were reported [57] and are indicated by the presence of decimal degrees of freedom. The η^2^_p_ effect size was calculated, and the assumed reference values were as follows: “small” effect = 0.01, “medium” effect = 0.06, and “large” effect = 0.14 [58]. Bonferroni-adjusted post hoc tests followed the repeated measures analyses to analyze pairwise comparisons. To determine effect sizes for significant pairwise interaction, independent and paired t-tests were used, assuming Cohen’s d reference values [59] as follows: “small” effect = 0.2, “medium” effect = 0.5, and “large” effect = 0.8. Statistical analyses were conducted in IBM SPSS Statistics version 27.

## 3. Results

All participants completed the intervention and post-testing, with no dropouts or adverse events reported.

All variables in both groups presented normally distributed data, after performing a Shapiro–Wilk test, with *p* > 0.05.

### 3.1. Descriptive Trends in Motor Performance

**Figure 1:**
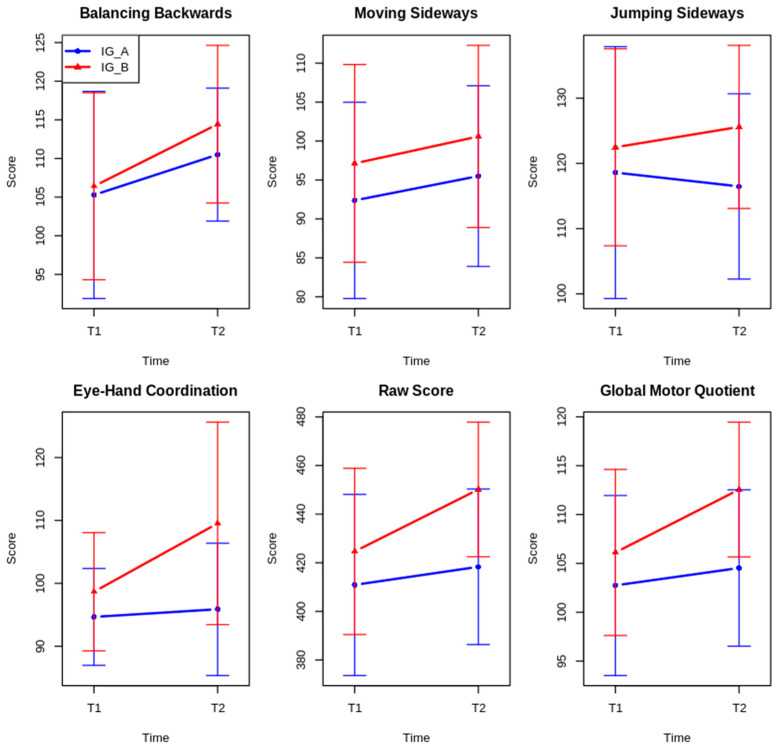
Interactive effects of group and time on fundamental motor skills. Note. IG_A = Intervention Group A; IG_B = Intervention Group B. Note. Error bars represent ±1 standard deviation.

Table 1 and Figure 1 present the pre- and post-intervention results for all motor variables, showing distinct patterns of change between groups. Intervention Group B (IG_B) demonstrated substantial improvements in Eye–Hand Coordination (increasing from 98.66 ± 9.4 to 109.53 ± 16.1), Raw Score (from 424.66 ± 34.2 to 450.16 ± 27.7), and Global Motor Quotient (from 106.12 ± 8.5 to 112.56 ± 6.9). In contrast, Intervention Group A (IG_A) showed more modest gains in these variables (EHC: 94.66 ± 7.7 to 95.88 ± 10.5; RS: 410.91 ± 37.2 to 418.34 ± 32.0; GMQ: 102.75 ± 9.2 to 104.53 ± 8.0). For fundamental motor skills, both groups improved in Balancing Backwards (IG_A: 105.28 ± 13.4 to 110.50 ± 8.6; IG_B: 106.41 ± 12.1 to 114.44 ± 10.2) and Moving Sideways (IG_A: 92.38 ± 12.6 to 95.50 ± 11.6; IG_B: 97.13 ± 12.7 to 100.59 ± 11.7), with IG_B achieving higher final scores. Jumping Sideways performance remained relatively stable in both groups.

### 3.2. Main Effects, Interactions, and Effect Size Interpretation

**Figure 2:**
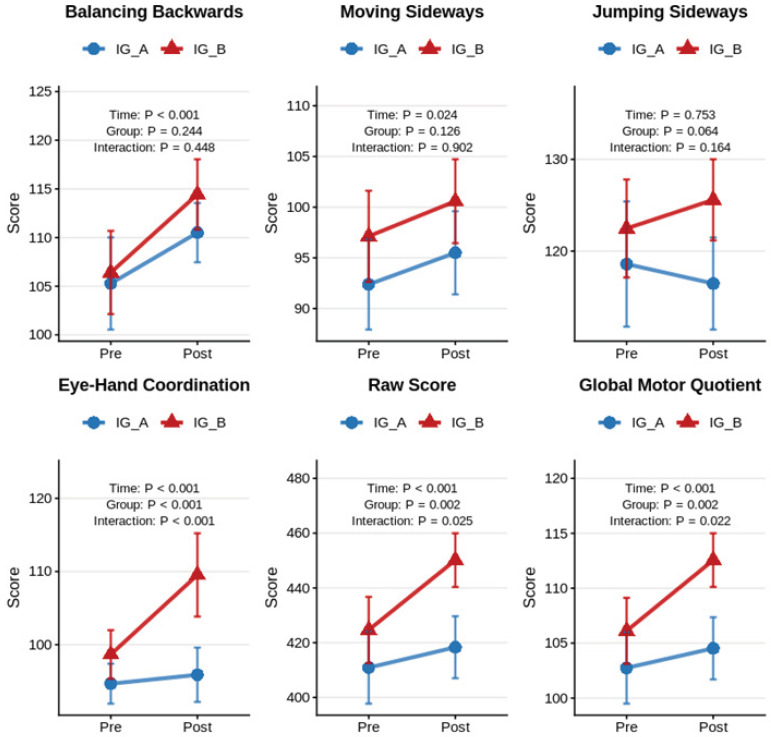
Effects of Intervention Approaches on Motor Competence: Changes in KTK3+ Global and Subtest Scores from Baseline to Follow-up. Note. Blue circles ● = Intervention Group A; Red triangles ▲ = Intervention Group B. Statistical significance levels are based on repeated-measures ANOVA; *p* < 0.001: Highly significant; *p* < 0.01: very significant; *p* < 0.05: Significant; NS: Not significant (*p* ≥ 0.05).

Figure 2 illustrates the pre–post-intervention changes in KTK3+ subtest scores for both intervention groups, while Table 2 presents the complete statistical analysis of these effects. Following conventional benchmarks for partial eta-squared (η^2^_p_), effects were interpreted as small (≥0.01), medium (≥0.06), or large (≥0.14).

As visually depicted in Figure 2 and supported by the statistical analysis in Table 2, Balancing Backwards (BB) and Moving Sideways (MS) showed significant main effects of time with large (BB: F(1,31) = 24.337, *p* < 0.001, η^2^_p_ = 0.440) and medium-to-large (MS: F(1,31) = 5.647, *p* = 0.024, η^2^_p_ = 0.154) effect sizes. The parallel improvement patterns visible in Figure 2 for both groups in these subtests align with the non-significant group effects and time × group interactions observed in Table 2 (η^2^_p_ = 0.044 and 0.019 for BB; η^2^_p_ = 0.074 and 0.001 for MS), suggesting comparable improvements with both intervention approaches. Bonferroni-adjusted pairwise comparisons confirmed that both groups showed significant pre-to-post improvements in these tasks.

In contrast, Jumping Sideways (JS) revealed no significant main effects or interactions in Table 2, with small effect sizes (Time: η^2^_p_ = 0.003; Group: η^2^_p_ = 0.107; Interaction: η^2^_p_ = 0.061), a pattern reflected in the minimal changes observed between pre- and post-intervention scores in Figure 2. Bonferroni-adjusted analyses confirmed the absence of significant pairwise differences.

The most notable findings emerged for Eye–Hand Coordination (EHC), where Figure 2 clearly shows the differential improvement pattern between groups. This visual pattern is statistically confirmed in Table 2, which demonstrates significant main effects of Time (F(1,31) = 25.327, *p* < 0.001, η^2^_p_ = 0.450), Group (F(1,31) = 14.003, *p* < 0.001, η^2^_p_ = 0.311), and their interaction (F(1,31) = 12.272, *p* < 0.001, η^2^_p_ = 0.284), all with large effect sizes. Bonferroni-adjusted pairwise comparisons revealed that only the IG_B group significantly improved from pre- to post-intervention (*p* < 0.001; Cohen’s d = 0.84), while IG_A showed no significant change. Significant between-group differences were observed at both time points, being more pronounced post-intervention (*p* < 0.001; Cohen’s d = 1.00) than pre-intervention (*p* = 0.034; Cohen’s d = 0.46).

Similarly, for the composite scores, both Raw Score (RS) and Global Motor Quotient (GMQ) in Figure 2 show a pattern where both groups improved, but IG_B demonstrated substantially greater gains. Table 2 confirms this with significant main effects of Time (RS: F(1,31) = 26.494, *p* < 0.001, η^2^_p_ = 0.461; GMQ: F(1,31) = 26.304, *p* < 0.001, η^2^_p_ = 0.459), Group (RS: F(1,31) = 10.838, *p* = 0.002, η^2^_p_ = 0.259; GMQ: F(1,31) = 10.906, *p* = 0.002, η^2^_p_ = 0.260), and Time × Group interactions (RS: F(1,31) = 5.537, *p* = 0.025, η^2^_p_ = 0.152; GMQ: F(1,31) = 5.808, *p* = 0.022, η^2^_p_ = 0.158), with medium to large effect sizes. Bonferroni-adjusted pairwise comparisons indicated that significant between-group differences were present only at the post-intervention time point (*p* < 0.001 for both RS and GMQ), with large Cohen’s d effect sizes (1.581 for RS and 1.595 for GMQ).

## 4. Discussion

This study investigated the effects of a 12-week training program focused exclusively on athletics vs. a combined program integrating athletics with a variety of motor sports experiences in children aged 6–10 years. The findings provide a valuable contribution to the growing literature on early MC development and offer nuanced insights into how distinct components of MC respond to structured physical interventions [24,60].

As reported in the results section, in the EHC test, only IG_B demonstrated significant pre- to post-intervention improvement. Although IG_B already presented higher baseline performance, these differences between groups became more pronounced post-intervention. This aligns with previous findings showing that tasks requiring perceptual–motor integration benefit from diverse and cognitively engaging practice contexts [12]. The inclusion of manipulative tasks and games in the multiactivity group may have enhanced sensorimotor integration processes, consistent with evidence suggesting that cognitively demanding motor experiences can enhance MC [24]. Similar patterns were observed for the composite KTK3+ results (RS and GMQ), which confirmed the added value of diversified motor experiences for global motor proficiency [10,61]. IG_B displayed significant pre- to post-test improvements in both RS and GMQ, while between-group differences emerged only post-intervention, indicating comparable baseline performance.

These findings reinforce the hypothesis that multicomponent programs, especially those combining athletic elements (running, jumping, coordination tasks) with varied motor activities (object control, balance, spatial orientation), may be particularly effective in promoting overall MC compared with traditional athletics programs [2,36,37,40,62]. Nevertheless, these results are specific to the present sample and its context, namely the IG_B superiority on EHC baseline values, and should, therefore, be evaluated with caution, as will be stressed later on in the study limitations.

Conversely, as expected and expressed in the hypotheses, both groups improved similarly in BB and MS performance, suggesting that the additional multiactivity content in IG_B neither enhanced nor impaired outcomes in these tests. A plausible explanation lies in these tasks, which were practiced in both intervention protocols.

The absence of significant effects for JS was unexpected, given that jumping and lateral movements are fundamental to both athletics and multiactivity sports. Nonetheless, this result may be explained by previous research indicating that gross motor tasks involving explosive power and rhythm may require longer or higher-intensity training to produce measurable improvements in children [11,27]. As we reinforce later on, the interventions may have been successful in enhancing coordination, an important factor underlying MC, but not in power-based motor skills. These skills may also be more sensitive to maturational changes or may demand task-specific reinforcement to elicit significant adaptations [30]. Supporting this view, two studies [32,63] showed that plyometric-based games can effectively enhance jumping ability and muscular power in this age group, an approach that could complement multiactivity interventions to target power-dominant tasks like JS. Furthermore, JS is a task where (theoretically) there are no ceiling effects, as it is always possible to improve performance, unlike BB, where 72 is the maximum absolute number of steps. However, it might be the case that the groups’ performance was already too high, leaving reduced space for improvement. In fact, that was a task where baseline results would fall under the “Good MC” category, while all the others would fall into “Normal MC”. Therefore, although there would be enough margin for progress, that was the task that had less space for improvement. Thus, and linking to the possibilities raised above, these may be some of the reasons for the lack of significant progress in their results during the intervention.

The differential impact of the intervention across variables reflects the principle of task specificity, which posits that motor skills adapt in distinct ways depending on training type, intensity, and structure [64]. The multiactivity intervention may have been especially effective in enhancing coordination and integrative aspects of MC, while exerting less influence on isolated locomotor or power-oriented skills.

As postulated in the hypotheses, the intervention did not produce significant interaction effects for certain variables (BB, MS, and JS). Several factors may explain the absence of differential gains between groups: Training specificity: Improvements in motor tasks are greatest when the practiced skills closely resemble the assessed skills [65]. As programs presented no significant differences in balance and specific locomotor tasks, substantial group differences were not expected. Moreover, skills such as JS may, as stressed before, rely heavily on anaerobic power, rhythm, and reactive strength, which are less responsive to general motor programs and may require targeted plyometric or high-load training [66]; although the MC assessment tool used in the present study is practical and widely used, it may lack the granularity needed to detect small but meaningful improvements, particularly in dynamic or rhythm-based motor skills. Future studies could incorporate motion capture or wearable sensor-based kinematic assessments to enhance sensitivity and detect subtle changes in performance. Additionally, KTK3+, like most of the MC assessment batteries, prioritizes product-oriented measures. Despite their ease of scoring, process-oriented assessments (e.g., TGMD-3 [67]) may be more sensitive for the detection of differences between stages of skill development, which may not be evident on MC product-based measures.

Revisiting hypotheses:

1. Both groups would show significant improvements over time in balance and locomotor-related tasks (BB, MS, JS): this hypothesis was confirmed for BB and MS but not for JS, as detailed before.
2. The multiactivity group (IG_B) would demonstrate greater improvements in the manipulative task—EHC: this hypothesis was confirmed. In fact, the IG_B group was the only group where improvements reached statistical significance.
3. Global MC indicators (RS and GMQ) would improve more in the multiactivity group compared with the athletics-only group: this hypothesis was confirmed. Again, the IG_B group was the only group where improvements reached statistical significance.

### Limitations, Strengths, and Future Research

While the study’s quasi-experimental design and the use of validated motor competence assessment tools strengthen internal validity, several limitations must be acknowledged.

Firstly, participants were all federated track-and-field athletes from a single club, which introduces several limitations: possible specificities of the club, facilities, participants’ MC entry level, and coaches’ interventions, among others, limit the generalizability of results and conclusions to other athletics environments or other sports environments. Thus, it is recommended that the study be replicated in other clubs to verify the trend identified in this research.

Additionally, the quasi-experimental design with ecological allocation based on parental schedule choice introduces a potential self-selection bias. In fact, some affinities between groups of parents and their children may have contributed to some clustering on the groups, either by residence, school attended, or other factors that might have intra-homogenized and, thus, differentiated the groups a priori.

The intervention duration (12 weeks) may have been relatively short to observe potential structural changes in some motor domains, particularly in strength, as reported by Sortwell et al. [63] and Nobre et al. [68].

Individual variability in prior motor experience (namely, training history), motivation, and cognitive engagement was not considered, although such factors are known to mediate motor learning outcomes [69,70]. In addition, the initial differences (significant only for EHC) between groups may have been a factor influencing the results. Thus, future studies should seek to ensure initial between-groups homogeneity, especially regarding EHC. In fact, group B, having superiority on EHC at baseline, may have had better initial conditions to progress more on that same variable over time, although this is something that cannot be confirmed or denied in the present study.

In addition, this study did not consider variables such as gender, socioeconomic status, or psychological factors (e.g., enjoyment, self-efficacy), which could be included in future research. Future studies should explore longer interventions combining multiactivity with focused modules (e.g., plyometrics, dance, or exergames) to more precisely target locomotor and power domains [11,32], and integrate cognitive–motor tasks, as supported by recent exergaming studies [12]. In addition, as all IG_B group children followed the same program with identical complementary activities, it is not possible to determine whether the observed MC improvements were due to a specific activity or to the overall combination of tasks, an aspect that should be addressed in future research. Still, regarding the IG_B group, it was also not possible to determine to what extent the fact that several coaches were involved in the differentiated weekly training influenced the final MC result. In other words, the IG_B group may have had better results in EHC and in the overall KTK3+ result, not because of the different activities presented, but because of characteristics related to their intervention profile and prognostic variables, among others. Hence, the influence of the coaches’ intervention on the learning outcomes could not be controlled, which might constitute a bias to results’ interpretation. Therefore, in future studies, to further narrow down extraneous variables, it will be necessary to record the coaches’ intervention through an analysis of verbal communication and to adequately provide their training for reliability and homogeneous intervention, aligned with the intervention of the principal investigator on the athletics training.

Additionally, the long-term sustainability of verified motor competence gains was something that was not assessed in the present study. Future studies would benefit from a follow-through procedure after the intervention process.

Furthermore, given the important foundation that FMS represent for MC, the use of process-oriented measures, along with MC product-oriented measures, like was the case of the present study with KTK3+, could be suggested on future studies, aiming a more comprehensive, sensitive and refined evaluation.

Finally, athletic skill performance was not assessed in the present study, as the focus was to determine potential differences between programs in motor competence development. Nevertheless, this is an important aspect that future studies should consider, since athletic coaches aim to enhance motor competence without compromising sport-specific skills. Should future research confirm that athletic skills are not impaired by less specific training, it would support the inclusion of such complementary activities within athletics training programs.

## 5. Conclusions

This study revealed that a 12-week athletics-only intervention program (IG_A), as well as a multiactivity training program, combining athletics with diverse sport and motor activities (IG_B), can significantly enhance MC features in children aged 6–10 years. The latter (IG-B) produced meaningful gains in EHC, GMQ, and total RS, which exceeded those observed in the former (IG_A). For BB and MS, both programs induced similar significant improvements. Conversely, none of the programs induced significant progress on JS. Therefore, the combination of athletics with several other sports and motor activities seems to have induced deeper and broader improvements in federated track-and-field athletes’ MC. However, these results and their interpretation should be tempered by the various limitations that have been postulated, namely the potential a priori bias that may have been induced by the constitution of the groups and by the reference level in some characteristics of the MC.

Future research should investigate the longitudinal effects of such programs and examine the interplay between cognitive, motivational, and physical domains to further optimize children’s motor development trajectories.

## Figures and Tables

**Table 1:** Results for each KTK3+ subtest, the RS and GMQ by group and time.

Groups/ Variables	Time (Moments)	BB	MS	JS	EHC	RS	GMQ
1 (IG_A)	1	105.28 ± 13.4	92.38 ± 12.6	118.59 ± 19.3	94.66 ± 7.7	410.91 ± 37.2	102.75 ± 9.2
2 (IG_B)	1	106.41 ± 12.1	97.13 ± 12.7	122.47 ± 15.1	98.66 ± 9.4	424.66 ± 34.2	106.12 ± 8.5
1 (IG_A)	2	110.50 ± 8.6	95.50 ± 11.6	116.47 ± 14.2	95.88 ± 10.5	418.34 ± 32.0	104.53 ± 8.0
2 (IG_B)	2	114.44 ± 10.2	100.59 ± 11.7	125.59 ± 12.5	109.53 ± 16.1	450.16 ± 27.7	112.56 ± 6.9

Note. IG_A and IG_B = Intervention Groups; BB = Balancing Backwards; MS = Moving Sideways; JS = Jumping Sideways; EHC = Eye–Hand Coordination; RS = Raw Score; GMQ = Global Motor Quotient. Time 1 = Baseline (pre-intervention); Time 2 = Follow-up (post-intervention).

**Table 2:** Differences between groups in pre- and post-intervention.

KTK3+ Variables	Independent Variables	Mean Square	F	df1	df2	*p*	η^2^_p_	Pairwise Comparisons
BB	Time	2809.000	24.337	1	31	0.001 **	0.440	2 ≠ 1
	Group	102.516	1.413	1	31	0.244	0.044	NS
	Time * Group	126.56	0.591	1	31	0.448	0.019	NS
MS	Time	695.641	5.647	1	31	0.024 *	0.154	2 ≠ 1
	Group	387.598	2.472	1	31	0.126	0.074	NS
	Time * Group	1.891	0.016	1	31	0.902	0.001	NS
JS	Time	16.000	0.101	1	31	0.753	0.003	NS
	Group	676.000	3.697	1	31	0.064	0.107	NS
	Time * Group	441	2.098	1	31	0.164	0.061	NS
EHC	Time	2340.141	25.327	1	31	<0.001 **	0.450	2 ≠ 1
	Group	1246.973	14.003	1	31	<0.001 **	0.311	2 ≠ 1
	Time * Group	1491.891	12.272	1	31	0.001 **	0.284	2 ≠ 1
RS	Time	17,358.063	26.494	1	31	<0.001 **	0.461	2 ≠ 1
	Group	8303.766	10.838	1	31	0.002 **	0.259	2 ≠ 1
	Time * Group	5220.063	5.537	1	31	0.025 *	0.152	2 ≠ 1
GMQ	Time	1080.766	26.304	1	31	<0.001 **	0.459	2 ≠ 1
	Group	520.410	10.906	1	31	0.002 **	0.260	2 ≠ 1
	Time * Group	346.891	5.808	1	31	0.022 *	0.158	2 ≠ 1

Note. BB = Balancing Backwards; MS = Moving Sideways; JS = Jumping Sideways; EHC = Eye–Hand Coordination; RS = Raw Score; GMQ = Global Motor Quotient. F = ANOVA test statistic; df = degrees of freedom. In pairwise comparisons, “2 ≠ 1” indicates a significant difference (e.g., between Time 2 vs. Time 1, or Group 2 vs. Group 1). * *p* < 0.05, ** *p* < 0.01.

## Data Availability

The data that support the findings of this study are available from the corresponding author upon request.

## Abbreviations

The following abbreviations are used in this manuscript:

BB	Balancing Backwards
EHC	Eye–Hand Coordination
FMS	Fundamental Motor Skills
GMQ	Global Motor Quotient
IG_A	Athletics training group
IG_B	Athletics training + complementary activities group
JS	Jumping Sideways
KTK3+	Körperkoordinationstest für Kinder3 + EHC
MABC	Movement Assessment Battery for Children
MC	Motor Competence
MS	Moving Sideways
N	Number
PA	Physical Activity
RS	Raw Score

## Appendix A

**Table A1:** Intervention Group (IG_A) Program.

Week	Sports	Training Objective	Exercises Prescription	Volume
1	Athletics	Techniques and Speed	Main part: Running techniques (Skipping) and Speed: various starts: sitting, lying down…	60 min
	Athletics	Speed and Strength	Main part: Agility and coordination circuit and General physical condition circuit	60 min
	Athletics	Strength and Resistance	Main part: General physical condition circuit and Varied games to stimulate resistance	60 min
2	Athletics	Techniques and Speed	Main part: Hurdles training technique and running high speed with 4, 5 and 6 low hurdles	60 min
	Athletics	Speed and Strength	Main part: Agility and coordination circuit and multi-throw with medicine ball (1–2 kg)	60 min
	Athletics	Strength and Resistance	Main part: General physical condition circuit and aerobic running (10–15 min)	60 min
3	Athletics	Techniques and Speed	Main part: Running technique training with cones and pins and Speed: Speed games	60 min
	Athletics	Speed and Strength	Main part: Fast running over short distances and multi-jumps to the sandpit	60 min
	Athletics	Strength and Resistance	Main part: Strength training on the stairs and Resistance games (Example: Formula 1)	60 min
4	Athletics	Techniques and Speed	Main part: Race Modeling (pins at different distances) Speed: Short-duration speed.	60 min
	Athletics	Speed and Strength	Main part: Pursuit race and Hurdles races; General physical condition circuit	60 min
	Athletics	Strength and Resistance	Main part: General physical condition circuit and Resistance games (Example: Suicide)	60 min
5	Athletics	Techniques and Speed	Main part: Long Jump techniques training and Speed: Short-duration speed (15 to 20 m)	60 min
	Athletics	Speed and Strength	Main part: Block star training and strength training on hill (fast running on short hills)	60 min
	Athletics	Strength and endurance	Main part: General physical condition circuit and aerobic running (10–15 min)	60 min
6	Athletics	Techniques and Speed	Main part: Block start training and race modeling; Speed: various starts: sitting, lying…	60 min
	Athletics	Speed and Strength	Main part: Agility and coordination circuit and General physical condition circuit	60 min
	Athletics	Strength and endurance	Main part: Agility and coordination circuit and long jump and Resistance games (Relay)	60 min
7	Athletics	Techniques and Speed	Main part: Relay training techniques and circuit of agility and coordination (slalom)	60 min
	Athletics	Speed and Strength	Main part: Various starts (sitting, lying down) and Strength training on the stairs	60 min
	Athletics	Strength and endurance	Main part: Strength training using only body weight and aerobic running (10–15 min)	60 min
8	Athletics	Techniques and Speed	Main part: High jump training and Speed: Short-duration speed (15 to 20 m)	60 min
	Athletics	Speed and Strength	Main part: Race Modeling (pins 3- or 4-m distance) Strength training—body weight	60 min
	Athletics	Strength and endurance	Main part: General physical condition circuit and Resistance games (Ex: Relay 4 × 400 m)	60 min
9	Athletics	Techniques and Speed	Main part: Running techniques (Skipping’s) and Speed: Speed Games (Ex: Divisions Game)	60 min
	Athletics	Speed and Strength	Main part: Short-duration speed (15–20 m) and Strength training with medicine balls	60 min
	Athletics	Strength and endurance	Main part: Multi-jumps to the sandpit and aerobic running (10–15 min)	60 min
10	Athletics	Techniques and Speed	Main part: Running technique training with cones and Agility and coordination circuit	60 min
	Athletics	Speed and Strength	Main part: Speed Games (Slalom and relay 4 × 40 m), Multi-throwing with medicine balls	60 min
	Athletics	Strength and endurance	Main part: Strength training using only body weight and aerobic running (15–20 min)	60 min
11	Athletics	Techniques and Speed	Main part: High jump training and Speed: Short-duration speed (30 to 40 m)	60 min
	Athletics	Speed and Strength	Main part: Speed (Relay 4 × 40 m) Strength Multi-jumps (boxes, ropes, stairs, hurdles, etc.)	60 min
	Athletics	Strength and endurance	Main part: General physical condition circuit and Resistance games (Ex: Formula 1)	60 min
12	Athletics	Techniques and Speed	Main part: Hurdles training technique and running high speed with 4, 5 and 6 low hurdles	60 min
	Athletics	Speed and Strength	Main part: Agility and coordination circuit and General physical condition circuit	60 min
	Athletics	Strength and endurance	Main part: Strength training using only body weight and aerobic running (15–20 min)	60 min

## Appendix B

**Table A2:** Intervention Group (IG_B) Program.

Week	Sports	Training Objective	Exercises Prescription	Volume
1	Athletics	Techniques and Speed	Main part: Running techniques (Skipping) and Speed: various starts: sitting, lying down…	60 min
	Gymnastic	Technique and initiation	Main part: Basic fundamentals of Gymnastics (Learning basic posture exercises)	60 min
	Athletics	Strength and Resistance	Main part: General physical condition circuit and Varied games to stimulate resistance	60 min
2	Athletics	Techniques and Speed	Main part: Hurdles training technique and running high speed with 4, 5 and 6 low hurdles	60 min
	Handball	Technique and initiation	Main Part: Relationship with the ball and fundamental (passing, receiving and shooting)	60 min
	Athletics	Strength and Resistance	Main part: General physical condition circuit and aerobic running (10–15 min)	60 min
3	Athletics	Techniques and Speed	Main part: Running technique training with cones and pins and Speed: Speed games	60 min
	M. Games	Technique and initiation	Main Part: Games to develop general MC (rhythm and co-operation games)	60 min
	Athletics	Strength and Resistance	Main part: Strength training on the stairs and Resistance games (Example: Formula 1)	60 min
4	Athletics	Techniques and Speed	Main part: Race Modeling (pins at different distances) Speed: Short-duration speed.	60 min
	Swimming	Technique and training	Main part: Learning basic aquatic motor skills (Free Games) and basic fundamentals of swimming.	60 min
	Athletics	Strength and Resistance	Main part: General physical condition circuit and Resistance games (Example: Suicide)	60 min
5	Athletics	Techniques and Speed	Main part: Long Jump techniques training and Speed: Short-duration speed (15 to 20 m)	60 min
	Handball	Technique and training	Main Part: Relationship with the ball (dribbling and feinting)	60 min
	Athletics	Strength and endurance	Main part: General physical condition circuit and aerobic running (10–15 min)	60 min
6	Athletics	Techniques and Speed	Main part: Block start training and race modeling; Speed: various starts: sitting, lying…	60 min
	M. Games	Technique and training	Main Part: Games with varied movements (Station: hopping, running, jumping, throwing)	60 min
	Athletics	Strength and endurance	Main part: Agility and coordination circuit and Long jump and Resistance games (Relay)	60 min
7	Athletics	Techniques and Speed	Main part: Relay training techniques and circuit of agility and coordination (slalom)	60 min
	Handball	Technique and training	Main Part: Relationship with the ball (dribbling and feinting)	60 min
	Athletics	Strength and endurance	Main part: Strength training using only body weight and aerobic running (10–15 min)	60 min
8	Athletics	Techniques and Speed	Main part: High jump training and Speed: Short-duration speed (15 to 20 m)	60 min
	Gymnastic	Technique and training	Main part: Basic fundamentals of gymnastics (Improving Jumping and rolling exercises)	60 min
	Athletics	Strength and endurance	Main part: General physical condition circuit and Resistance games (Ex: Relay 4 × 400 m)	60 min
9	Athletics	Techniques and Speed	Main part: Running techniques (Skipping’s) and Speed: Speed Games (Ex: Divisions Game)	60 min
	M. Games	Technique and training	Main part: Object manipulation games and balance challenges with balls, hoops, ropes.	60 min
	Athletics	Strength and endurance	Main part: Multi-jumps to the sandpit and aerobic running (10–15 min).	60 min
10	Athletics	Techniques and Speed	Main part: Running technique training with cones and Agility and coordination circuit	60 min
	Swimming	Technique and training	Main Part: Learning basic aquatic motor skills (Propulsion and manipulation exercises)	60 min
	Athletics	Strength and endurance	Main part: Strength training using only body weight and aerobic running (15–20 min)	60 min
11	Athletics	Techniques and Speed	Main part: High jump training and Speed: Short-duration speed (30 to 40 m)	60 min
	Handball	Technique and training	Relationship with the ball and fundamental technical and tactical notions in game.	60 min
	Athletics	Strength and endurance	Main part: General physical condition circuit and Resistance games (Ex: Formula 1)	60 min
12	Athletics	Techniques and Speed	Main part: Hurdles training technique and running high speed with 4, 5 and 6 low hurdles	60 min
	M. Games	Technique and training	Main part: Circuit of games for cooperation, manipulation, locomotion and coordination.	60 min
	Athletics	Strength and endurance	Main part: Strength training using only body weight and aerobic running (15–20 min)	60 min
